# Niagara University Medical College

**Published:** 1891-05

**Authors:** 


### NIAGARA UNIVERSITY MEDICAL COLLEGE.

THE annual commencement exercises of the medical department of Niagara University occurred on Tuesday, April 14, 1891.

The Alumni Association held its annual meeting in the morning at the college building. The address of welcome on behalf of the faculty was delivered by Prof. Floyd S. Crego, M. D., who, in fitting words, traced the onward march of the school and the progress accomplished and to be accomplished. The dedication of the new building, and the establishments of new chairs and larger clinical facilities, were looked upon as most auspicious. Dr. Crego, in the course of his address, spoke approvingly of the new law providing for State examination and license. The response on behalf of the alumni was made by Dr. D. L. Redmond, President of the Association. The election of officers for the ensuing year resulted as follows:

*President*, J. J. Twohey, M. D.; *Vice-President*, E. A. Forsyth, M. D.; *Second Vice-President*, A. J. Colton, M. D.; *Secretary*, W. H. Norrish, M. D.; *Treasurer*, H. D. Ingraham, M. D.; *Executive Committee*, E. A. Smith, M. D., M. J. O'Connell, M. D., E. N. Pfohl, M. D.

Doctors William C. Krauss and L. Bradley Dorr, of the faculty, were elected to active, and Dr. Charles S. Evans, of Cincinnati, to honorary memberships.

The afternoon session was devoted to the reading and discussion of papers.

Dr. William C. Krauss, of Buffalo, read a paper entitled, On the Behavior of Tendon Reflexes, in Health and Disease.

The second paper, On Bacteria and their Relation to Certain Diseases, was presented by Dr. Charles Seth Evans, of Cincinnati, Ohio. Both papers were interesting and instructive, and held the attention of those present.

In the evening the commencement exercises were held at the Star Theater, and the following named graduates were placed upon the record books as Doctors of Medicine: Jacob Frederick Meyer, Buffalo, N. Y.; Daniel Francis Sullivan, Hartford, Conn.; Thomas Joseph Burke, Newburg, N. Y.; Franklin Charles Gram, Buffalo, N. Y.; Francis William Maloney, Niagara Falls, N. Y.; Robert Andrew Poynton, Greenwood, N. Y.; Jacob Smith Peterson, Buffalo, N. Y.; William Frederick Beutler, Buffalo, N. Y.; John David Howland, Buffalo, N. Y. The commencement address was given by Dr. Simeon Tucker Clark, of Lockport. Immediately following these exercises many proceeded to The Genesee, where the Alumni banquet was held. Thus closed an important and successful year in the history of the Niagara Medical College.

The Spring session began April 20, 1891, to continue six weeks, and is well attended.

